# Myocardial Injuries in COVID-19: More Questions Than Answers

**DOI:** 10.3390/jcm11154527

**Published:** 2022-08-03

**Authors:** Alfredo Bardaji

**Affiliations:** 1 Department of Cardiology, Joan XXIII University Hospital, 43005 Tarragona, Spain; abardaji.hj23.ics@gencat.cat; 2 Department of Medicine and Surgery, Rovira i Virgili University, 43003 Tarragona, Spain; 3 Pere Virgili Health Research Institute (IISPV), 43005 Tarragona, Spain

At the end of 2019, the SARS-CoV-2 virus was reported to be responsible for the cases of pneumonia that had begun to appear a few months earlier in the Wuhan province of China. This was a new strain of coronavirus that had not been previously identified in humans and caused the disease known as COVID-19. The worldwide spread of this virus was explosive: A few months later, in May 2020, 3.67 million people had been infected and more than 250,000 had died. Two years later, in May 2022, more than 522 million confirmed cases had been reported, with over six million deaths worldwide. In the first two and a half years of the pandemic, we have learned substantially more about this infection and how it interacts with the cardiovascular system [1]. We also know that COVID-19 confers significant cardiovascular morbidity and mortality in patients with or without prior cardiovascular disease. We know that cardiovascular manifestations in COVID-19 are very varied and include myocarditis, acute coronary syndrome, heart failure, cardiogenic shock, arrhythmias, and thromboembolic and cerebrovascular complications [2].

Many cardiovascular manifestations in COVID-19 have the early detection of myocardial injury in common, which is diagnosable by elevated cardiac troponin (cTn), a fundamental biomarker in cardiovascular research in the diagnosis and prognosis of patients with acute coronary syndrome. Approximately 10–20% of patients hospitalised for COVID-19 have evidence of myocardial injury [3]. We know that cTn elevation as an indicator of myocardial injury is very frequent in any acute pathology, especially critical or infectious processes [4]. Troponin elevation in SARS-CoV-2 infection currently still has many unknowns. The first question is whether the myocardial injury detected in patients with COVID-19 is specific or similar to that which can be seen in any other serious diseases treated in an Emergency Department [5].

Direct myocardial injury to cardiomyocytes by the virus itself is possible. However, cases of myocarditis in COVID-19 infection are rare [6], less symptomatic than myocarditis in patients without COVID-19, and associated with higher hospital mortality. In general, the mechanisms that cause myocardial injury in COVID-19 can be very varied: for example, cardiac toxicity induced by ACE2, immune and inflammatory response; mitochondrial dysfunction and oxidative stress; endothelial injury, hypoxemia, or autonomic imbalance [7].

The interpretation of a troponin elevation in patients with COVID-19 is a great challenge for the Emergency Department. On the one hand, it is always necessary to rule out an acute coronary syndrome (type 1 infarction) based on data from the clinical history and the electrocardiogram. However, type 1 infarctions are very rare in patients with COVID-19. Therefore, troponin elevation in these patients must be interpreted as acute ischemic myocardial injury (type 2 infarction) or non-ischemic myocardial injury. For the diagnosis of type 2 myocardial infarctions, it is necessary to investigate a clinical situation associated with an imbalance between the supply and consumption of oxygen by the myocardium. This situation is not uncommon in critically ill patients with a COVID-19 infection, who are often tachycardic, hypotensive, or hypoxemic [3]. Proposed mechanisms for this myocardial damage include the activation of inflammatory and thrombotic cascades, direct viral damage to the myocyte or vascular endothelium, and worsening basal atherosclerotic or structural abnormalities [8].

The distinction between type 2 myocardial infarction and non-ischemic acute myocardial injury may not be clinically relevant. Both cases are associated with a similar and poor prognosis, which is fundamentally determined by the severity of the systemic respiratory involvement of infection by the SARS-CoV-2 virus. Furthermore, the detection of dynamic changes between two troponin determinations, regardless of the presence of myocardial damage (Tn max > 99th), is also associated with a worse prognosis [9]. In some cases, myocardial injury is possible before COVID-19 infection and is conditioned by previous cardiovascular involvements, which are one of the main factors for a poor prognosis [1,10]. The correlation between myocardial injuries detected by troponin elevation and myocardial injury detected by imaging techniques such as cardiac resonance is also controversial. Abnormalities have been described in cardiac magnetic resonance performed in the subacute phase of the disease in patients in whom troponin elevations had not been detected in an acute phase, which is somewhat surprising [11].

However, we do know that the short-term cardiovascular effects on the long-term consequences in COVID-19 patients are related to myocardial injuries detected in the acute phase [12,13]. It is essential to recognise that, in some cases, a myocardial injury will persist once the acute phase of the infection has passed [14]. The long-term implication of this chronic myocardial injury is still poorly understood. The development of new ventricular dysfunction during follow-up is believed to be related to myocarditis, microvascular or endothelial injury, and myocardial stress due to an imbalance as a result of increased myocardial demand and decreased myocardial oxygenation due to hypoxia, myocardial inflammation, or proinflammatory cytokines occurring in the acute phase. All of these are processes associated with myocardial injury. However, the prediction of ventricular dysfunction in follow-up is something that we still know little about. Fortunately, long-term cardiac imaging studies after COVID-19 infection show very few cardiovascular sequelae in most patients [15]. Those patients whose disease persists after the first 4 weeks after infection often have cardiovascular manifestations such as chest pain, palpitations, inappropriate sinus tachycardia, postural orthostatic tachycardia syndrome, atrial arrhythmias, cardiomyopathy, and thromboembolism.

The evolution of different variants in the SARS-CoV-2 virus and the generalisation of vaccination guidelines in most Western countries may also change cardiovascular involvement in COVID-19 and, therefore, the presence of myocardial injury [8,16]. An interesting aspect is that patients immunised against influenza had lower risks of hospitalisation and ICU admission against COVID-19 than those who are not vaccinated [17].

In conclusion, myocardial injury in COVID-19 is frequent and is associated with a poor prognosis. Currently, we have many unanswered questions, which is why an intense research program is necessary for this area to better understand an infection that will accompany us in the coming years.

## References

[1] Núñez-Gil I.J., Fernández-Ortiz A., Eid C.M., Huang J., Romero R., Becerra-Muñoz V.M., Uribarri A., Feltes G., Trabatoni D., Fernandez-Rozas I. (2021). Underlying heart diseases and acute COVID-19 outcomes. Cardiol. J..

[2] Alqahtani M., Abbas M., Alsabaani A., Alqarni A., Almohiy H.M., Alsawqaee E., Alshahrani R., Alshahrani S. (2022). The potential impact of COVID-19 virus on the heart and the circulatory system. Infect. Drug Resist..

[3] Bozkurt B., Das S.R., Addison D., Gupta A., Jneid H., Khan S.S., Koromia G.A., Kulkarni P.A., LaPoint A., Lewis E.F. (2022). 2022 AHA/ACC key data elements and definitions for cardiovascular and non-cardiovascular complications of COVID-19. J. Am. Coll. Cardiol..

[4] Bardají A., Cediel G., Carrasquer A., De Castro R., Sánchez R., Boqué C. (2015). Troponin elevation in patients without acute coronary syndrome. Rev. Esp. Cardiol..

[5] Bardají A., Carrasquer A., Sánchez-Giménez R., Lal-Trehan N., Del-Moral-Ronda V., Peiró Ó.M., Castilho G., Fort-Gallifa I., Benavent C., Recio G. (2021). Prognostic implications of myocardial injury in patients with and without COVID-19 infection treated in a university hospital. Rev. Esp. Cardiol..

[6] Mirò Ò., Sabaté M., Jiménez S., Mebazaa A., Martínez-Nadal G., Piñera P., Burillo-Putze G., Martín A., Martín-Sánchez F.J., Jacob J. (2022). A case-control, multicentre study of consecutive patients with COVID-19 and acute (myo)pericarditis: Incidence, risk factors, clinical characteristics and outcomes. Emerg. Med. J..

[7] Xu S., Wu W., Zhang S. (2022). Manifestations and mechanism of SARS-CoV-2-mediated cardiac injury. Int. J. Biol. Sci..

[8] Chatterjee A., Saha R., Mishra A., Shilkar D., Jayaprakash V., Sharma P., Sarkar B. (2022). Molecular determinants, clinical manifestations and effects of immunisation on cardiovascular health during COVID-19 pandemic era-A review. Cur. Probl. Cardiol..

[9] Polcwiartek C., Krogager M.L., Andersen M.P., Butt J.H. (2020). Prognostic implications of serial high-sensitivity cardiac troponin testing among patients with COVID-19: A Danish nationwide registry-based cohort study. Am. Heart J. Plus.

[10] Ng S.M., Pan J., Mouyis K., Kondapally Seshasai S.R., Kapil V., Rice K.M., Gupta A.K. (2022). Quantifying the excess risk of adverse COVID-19 outcomes in unvaccinated individuals with diabetes mellitus, hypertension, ischaemic heart disease or myocardial injury: A meta-analysis. Front. Cardiovasc. Med..

[11] Zhang L., Wei X., Wang H., Jiang R., Tan Z., Ouyang J., Li X., Lei C., Liu H., Liu J. (2022). Cardiac involvement in patients recovering from Delta Variant of COVID-19: A prospective multi-parametric MRI study. ESC Heart Fail..

[12] Li Y., Pei H., Zhou C., Lou Y., Lou Y. (2022). Myocardial injury predicts risk of short-term all-cause mortality in patients with COVID-19: A dose–response meta-analysis. Front. Cardiovasc. Med..

[13] Tobler D.L., Pruzansky A.J., Naderi S., Ambrosy A.P., Slade J.J. (2022). Length-term cardiovascular effects of COVID-19: Emerging data relevant to the cardiovascular clinician. Curr. Atheroscler. Rep..

[14] Lu J.Q., Lu J.Y., Wang W., Liu Y., Buczek A., Fleysher R., Hoogenboom W.S., Zhu W., Hou W., Rodriguez C.J. (2022). Clinical predictors of acute cardiac injury and normalisation of troponin after hospital discharge from COVID-19: Predictors of acute cardiac injury recovery in COVID-19. eBioMedicine.

[15] Gao Y.P., Zhou W., Huang P.N., Liu H.Y., Bi X.J., Zhu Y., Sun J., Tang Q., Li L., Zhang J. (2021). Normalised cardiac structure and function in COVID-19 survivors late after recovery. Front. Cardiovasc. Med..

[16] Alam L., Omar A.M.S., Talebi S., Narula J., Argulian E. (2022). Echocardiographic findings in patients with COVID-19 with myocardial injury during the Omicron variant surge. Am. J. Cardiol..

[17] Behrouzi B., Araujo Campoverde M.V., Liang K., Talbot H.K., Bogoch I.I., McGeer A., Fröbert O., Loeb M., Vardeny O., Solomon S.D. (2020). Influenza vaccination to reduce cardiovascular morbidity and mortality in patients with COVID-19: JACC State-of-the-Art Review. J. Am. Coll. Cardiol..

